# Wearable Sensor Technology for Hyperhidrosis Management in Individuals With Prosthetic Limbs: A Narrative Review

**DOI:** 10.7759/cureus.79109

**Published:** 2025-02-16

**Authors:** Kelly M Frasier, Mary Grace Hash, Andrew Pugliese

**Affiliations:** 1 Dermatology, Northwell Health, New Hyde Park, USA; 2 Dermatology, Edward Via College of Osteopathic Medicine, Auburn, USA; 3 Dermatology, Touro College of Osteopathic Medicine, Middletown, USA

**Keywords:** biosensor-based detection, biosensors, healthcare biosensors, prosthetic limb, smart health wearable, wearable biosensing devices, wearable devices, wearable medical devices

## Abstract

Wearable sensor technologies offer a cutting-edge solution for managing hyperhidrosis in individuals with prosthetic limbs, directly addressing the complex challenges posed by excessive sweating at the prosthetic-skin interface. Excessive moisture can lead to skin breakdown, increased risk of infections, and compromised prosthetic fit, all of which reduce functionality and user comfort. Advanced biosensors embedded within the system continuously monitor moisture levels and skin temperature in real time, providing precise data on sweat production and skin conditions. This data is relayed to mobile health platforms, allowing users and clinicians to make informed, immediate adjustments, such as modifying prosthetic materials, adjusting fit, or activating integrated cooling mechanisms to mitigate complications. Integrating real-time feedback into the device’s function offers a personalized, noninvasive approach, enhancing both comfort and long-term prosthetic performance while surpassing traditional hyperhidrosis treatments like systemic medications, topical therapies, or invasive interventions such as botulinum toxin injections. However, limitations such as sensor calibration issues in fluctuating environmental conditions, sensor durability, and ensuring user compliance remain challenges. Future advancements may incorporate machine learning algorithms to predict and preempt hyperhidrosis episodes, offering even more precise control and adaptability. With the potential for seamless integration into telemedicine platforms, wearable sensor technologies have the potential to revolutionize the management of hyperhidrosis for prosthetic users, offering a personalized, real-time solution that addresses both medical and functional challenges.

## Introduction and background

Hyperhidrosis, characterized by excessive sweating beyond physiological needs, significantly impacts the quality of life. While 2.8% of the general population experiences hyperhidrosis, studies suggest that more than 53% of individuals with amputations experience heat and/or perspiration discomfort inside their prostheses [1,2]. This highlights a significant issue that may contribute to hyperhidrosis or related complications in this specific population. Excessive sweating at the prosthetic-skin interface can lead to skin irritation, infections, and impaired prosthetic fit [2]. These complications not only cause physical discomfort but also reduce prosthetic functionality and limit mobility. Hyperhidrosis is often triggered by thermoregulatory factors, emotional stress, or underlying medical conditions, and its management in prosthetic users requires tailored approaches to address the unique interactions between skin and prosthetic materials [3]. Addressing hyperhidrosis in individuals with prosthetic limbs requires prioritizing the development of new treatments and innovations to improve their quality of life and overall well-being.

Managing hyperhidrosis in prosthetic users requires a nuanced approach, as current treatments often fall short of addressing the unique challenges faced by this population. Traditional options, including topical antiperspirants, systemic medications such as anticholinergics, and more invasive methods like botulinum toxin injections, generally target localized sweating but do not fully meet the needs of prosthetic users [4]. Treatments frequently offer inconsistent relief, leading to ongoing discomfort and increased risk of skin complications. Excessive sweating can cause prosthetic slippage, reduce suction adherence, and heighten the likelihood of bacterial or fungal infections at the skin-prosthetic interface [5]. There is an urgent need for innovative solutions that manage sweat production while enhancing skin health and improving prosthetic performance.

Wearable sensor technologies have emerged as a novel approach for managing hyperhidrosis. These technologies enable continuous monitoring of physiological parameters such as moisture levels and skin temperature. By integrating advanced biosensors into prosthetic devices, real-time feedback can be provided to both users and healthcare providers [6]. Sensors allow for immediate adjustments, such as modifying prosthetic fit or activating cooling mechanisms, to mitigate the negative effects of excessive sweating. Moreover, the data collected by these sensors can be transmitted to mobile health platforms, enabling more personalized and adaptive care tailored to the individual’s specific needs [6]. Unlike traditional treatments, which are often reactive and limited in scope, wearable sensor technologies offer a proactive, real-time solution that addresses the problem at its source.

As the technology evolves, there is significant potential to enhance these wearable sensors by incorporating machine learning algorithms capable of predicting hyperhidrosis episodes. Integrating machine learning and wearable sensors allows for pattern recognition, ultimately bridging the gap between analysis and desired outcomes [7]. Such advancements would enable even greater precision and adaptability, allowing the system to preemptively respond to changes in sweat production. Despite the promising outlook, challenges remain, including ensuring sensor durability, maintaining accuracy in various environmental conditions, and encouraging consistent user adherence [8]. However, with further development and integration into telemedicine platforms, wearable sensors for hyperhidrosis management could revolutionize care for individuals with prosthetic limbs, offering a comprehensive solution to both functional and medical challenges associated with excessive sweating.

Methods

A structured approach was employed to conduct a comprehensive narrative review of the literature on hyperhidrosis management in prosthetic users. The review process was designed to ensure transparency and an unbiased synthesis of evidence, in alignment with the best practices for narrative reviews. A comprehensive search of electronic databases, including PubMed, Embase, and Cochrane Library, was conducted to identify relevant studies published up to December 1, 2024. The search strategy combined keywords such as "hyperhidrosis", "prosthetic limbs", "prosthetic-skin interface", "wearable sensors", and "sweat management". Inclusion criteria encompassed peer-reviewed original research, clinical trials, systematic reviews, and relevant narrative reviews focusing on hyperhidrosis in prosthetic users. Studies addressing wearable sensor technologies for hyperhidrosis management were also included. Findings were synthesized thematically to identify current challenges in hyperhidrosis management for prosthetic users, evaluate the efficacy of existing treatments, and highlight innovative solutions such as wearable sensor technologies. Quantitative results were summarized using descriptive statistics, and qualitative data were analyzed to identify trends and gaps in the literature. To minimize bias, the review adhered to a prespecified protocol, and all decisions regarding inclusion, exclusion, and synthesis were documented. Conflicts of interest within included studies were critically evaluated, and the authors disclose no conflicts influencing this review.

## Review

The management of hyperhidrosis in individuals with prosthetic limbs represents a significant yet underexplored area of research that requires comprehensive attention, particularly considering the complexities associated with excessive sweating at the skin-prosthetic interface. Hyperhidrosis can manifest as a primary condition or as a secondary issue related to underlying medical conditions, which makes understanding its impact on prosthetic functionality and user comfort all the more critical [9]. Notably, a postal survey by Davidson revealed that 55% of limb amputees rated their limb sweating as “not acceptable,” a key factor contributing to low prosthetic use [9]. Existing treatments, such as topical antiperspirants and systemic medications, often fail to provide relief for prosthetic users, highlighting the urgent need for innovative strategies that address their unique demands [4]. Clinical cases have documented how excessive sweating can lead to skin irritation, discomfort, and even infections, ultimately impairing the usability and performance of prosthetic limbs [10]. Thus, a more nuanced approach that combines effective hyperhidrosis management with the optimization of prosthetic functionality is essential for improving outcomes in this population.

Emerging technologies offer promising strategies for managing hyperhidrosis in prosthetic users by integrating advanced biosensors for real-time sweat and temperature monitoring. Wearable electrochemical sensors, known for their affordability and high performance, continuously track key analytes such as sodium, potassium, and pH, making them valuable for managing sweat at the prosthetic interface [11]. Sensors with high levels of specificity and sensitivity offer crucial insights by monitoring sweat production and skin temperature, helping to prevent skin irritation and discomfort while enabling timely adjustments to enhance both comfort and prosthetic functionality. The Discovery Patch® (Epicore Biosystems, Cambridge, MA, USA) uses biosensors to monitor sweat and environmental conditions [12]. The system tracks hydration and electrolyte levels using soft, flexible microfluidic sensors that provide seamless integration with the skin [13]. Epicore Biosystems has also partnered with Gatorade to develop the Gx Sweat Patch®, designed to monitor athletes’ sweat rate and chloride concentration [14]. The technology utilized by these devices combines physiological data with app-based recommendations, facilitating adjustments to improve prosthetic comfort. While current systems focus on hydration, adapting them to detect hyperhidrosis could significantly expand their usefulness. Combining niche sensor technologies could provide a comprehensive solution for prosthetic users managing excessive sweating. 

The incorporation of artificial intelligence (AI) and machine learning into hyperhidrosis management systems represents another groundbreaking advancement in this field. Algorithms analyzing patterns in sweat production and user activity have the potential to predict hyperhidrosis episodes and recommend personalized interventions. For example, Sweat Buddy, a wearable device that integrates AI technology, monitors sweat levels and can alert users to imminent episodes. Similarly, machine learning models, such as neural networks and random forests, have shown high accuracy in predicting compensatory hyperhidrosis after surgical interventions like sympathectomy, enhancing personalized treatment outcomes [15]. In practical applications, such systems might suggest modifications to the prosthetic fit or activate built-in cooling mechanisms when significant sweating is detected. While recent systems and technologies show promise for shifting hyperhidrosis management from a reactive to a proactive model, variability in individual responses can lead to inaccuracies in predicting sweat events. This is often due to biases in the training data or differences between the model’s training population and real-world application, underscoring the need for further refinement in predictive algorithms [16]. While AI-powered solutions hold significant potential to transform hyperhidrosis management into a more proactive approach, ongoing refinements are essential to address the limitations posed by data variability and predictive accuracy, ensuring these technologies can be effectively integrated into everyday clinical practice. 

Beyond the physiological aspects, it is essential to consider the psychosocial implications of hyperhidrosis among prosthetic users. Excessive sweating can profoundly impact self-esteem and social interactions, often leading to feelings of embarrassment or isolation. Research indicates that individuals with hyperhidrosis experience higher rates of depression and anxiety compared to those without the condition [17]. Similarly, studies on prosthetic users have found that hyperhidrosis not only affects the physical fit of the prosthetic but also significantly contributes to social isolation and anxiety, as sweating issues interfere with daily activities and community participation [2]. To address these psychosocial challenges, future developments in wearable technologies should aim to connect users with support networks and educational resources, thereby promoting positive mental health alongside physical well-being. Wearable technologies that monitor both physical and mental well-being have already shown promise in addressing psychosocial challenges, as they provide real-time feedback and personalized support for users [18]. Devices that facilitate community engagement and provide access to information about hyperhidrosis management may empower users, helping them feel less isolated in their experiences. Integrating features such as chat support or access to online forums within these technologies could foster a sense of community and shared experiences among users, enhancing both emotional and social well-being.

Advancements in materials science also play a crucial role in addressing the unique challenges posed by hyperhidrosis in prosthetic users. Innovative materials like hydrophobic or moisture-wicking fabrics for prosthetic liners can significantly reduce sweat accumulation at the skin-prosthetic interface. A recent study demonstrated that moisture-wicking fabrics reduced skin irritation and enhanced comfort for prosthetic users [19]. Integrating these materials within prosthetic liners shows promise for allowing heat dissipation with greater regulation of sweat production. Furthermore, breathable, antibacterial fabrics help prevent bacterial growth and odors, improving hygiene and extending the liner’s lifespan [20]. Materials can be combined with smart textiles that incorporate biosensors for continuous monitoring, offering seamless technology integration without compromising user comfort [8,21]. Integrating such biosensors within the textile can alert users and clinicians of potential issues, thus allowing for more timely interventions and preventing the aforementioned complications. Utilizing a multifaceted approach emphasizes the importance of addressing both biological and mechanical aspects of hyperhidrosis management to enhance user experience and device efficacy.

Challenges related to accessibility and affordability remain critical considerations in the development of wearable sensor technologies. While these technologies show promise, ensuring their availability to diverse populations is essential for promoting equitable healthcare. Real-world implementations, such as those used in clinical trials, often highlight disparities in access to these innovations, particularly in underserved communities. For instance, a study found that low-income populations faced significant barriers to accessing wearable health monitoring devices due to high costs and lack of insurance coverage [22]. As stated by Cruz et al., with increased accessibility and affordability, there would be an increase in usage and compliance [22]. As such, individuals would have a more involved and sought-after approach to their well-being. Collaborative efforts among policymakers, manufacturers, and healthcare providers can bridge this gap, ensuring these innovations are more widely available. Educational programs aimed at healthcare professionals can also raise awareness regarding the importance of hyperhidrosis management for prosthetic users, leading to better patient outcomes.

Regulatory and ethical considerations must also be considered when discussing wearable sensor technologies. Since these devices collect sensitive health data, establishing robust data privacy and security frameworks becomes essential to ensure users feel confident that their information is protected. Without strict regulations, users may hesitate to adopt these innovations due to concerns about potential data breaches and misuse. Clear guidelines and transparency from manufacturers regarding data usage and storage are critical to fostering trust, especially given past healthcare data breaches [23]. Ethical issues related to informed consent for data collection must also be addressed, ensuring users fully understand how their data will be used for research and development purposes. 

**Figure 1:**
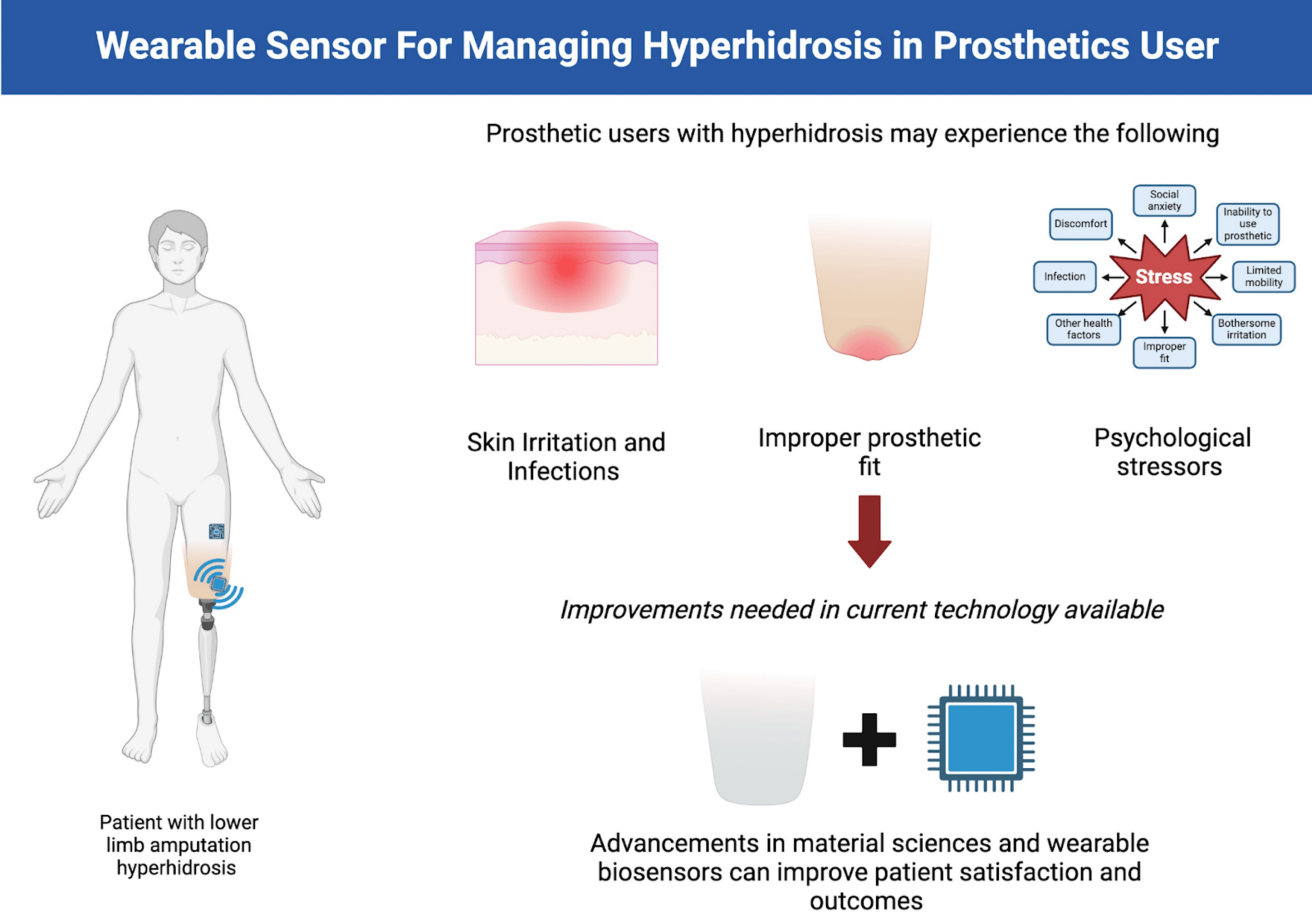
Illustration depicting common challenges faced by prosthetic users with hyperhidrosis, including skin irritation, improper prosthetic fit, and psychological stressors. The diagram highlights the need for advancements in materials science and wearable biosensor technology to improve patient satisfaction and outcomes. Image credit: Andrew Pugliese.

The intersection of wearable sensor technology and hyperhidrosis management presents a rich field for exploration, potentially improving the quality of life for individuals with prosthetic limbs who experience excessive sweating (Figure 1). Comprehensive solutions can emerge by integrating advancements in biosensors, AI, and materials science while also addressing the psychosocial factors associated with hyperhidrosis. Biosensors and AI, already prominent in clinical investigations, hold significant potential. For example, continuous glucose monitors not only provide patients with vital health data but also offer healthcare systems new research avenues and personalized approaches to medicine [24]. Ongoing research and collaboration among stakeholders will enhance prosthetic technology and support a holistic approach to physical and mental well-being in this unique population. Innovation in hyperhidrosis management for prosthetic users is timely, with the potential for transformative outcomes for both users and healthcare providers.

Strengths and limitations 

This study provides a comprehensive and multidisciplinary review of the challenges and emerging innovations in hyperhidrosis management for prosthetic users, addressing a significant gap in the existing literature. One of its key strengths lies in its approach to synthesizing evidence from diverse fields, including dermatology, prosthetics, materials science, and wearable sensor technology. By integrating clinical, technological, and psychosocial perspectives, this review presents a holistic framework for understanding hyperhidrosis management in this unique population. The inclusion of novel technologies, such as wearable biosensors and AI-driven predictive models, highlights innovative solutions while grounding them in real-world applicability. The review also emphasizes the importance of psychosocial well-being, drawing attention to how hyperhidrosis impacts mental health and social interactions, thereby broadening the scope of hyperhidrosis research beyond its physical manifestations. Additionally, the discussion on regulatory and ethical considerations ensures a forward-looking perspective on the challenges of implementation and data security, making the findings both timely and relevant for stakeholders, including researchers, clinicians, and policymakers.

However, this study has notable limitations. First, the heterogeneity of existing studies on hyperhidrosis and prosthetic users complicates direct comparisons and the generalizability of findings. The variability in study designs, sample sizes, and participant demographics limits the ability to draw definitive conclusions about the efficacy of current treatments and emerging technologies. Furthermore, much of the reviewed evidence is derived from early-stage or prototype-level innovations, such as wearable sensors and AI algorithms, which may not yet be validated in large-scale clinical trials. This introduces uncertainty regarding the feasibility, scalability, and cost-effectiveness of these solutions in real-world settings. Another limitation is the lack of specific data addressing hyperhidrosis in prosthetic users as a distinct subgroup, with much of the discussion extrapolated from broader studies on hyperhidrosis or wearable health technologies. Finally, while the review highlights the importance of accessibility and affordability, it does not include a detailed cost analysis or policy recommendations to address disparities in access to these innovations, which could hinder their widespread adoption. Despite these limitations, this study provides a critical foundation for advancing research and clinical practice in this underexplored area, serving as a call to action for further multidisciplinary collaboration and innovation.

## Conclusions

The integration of wearable sensor technologies into hyperhidrosis management represents a groundbreaking advancement with significant potential to improve the quality of life of individuals with prosthetic limbs. By employing sophisticated biosensors capable of continuously monitoring physiological parameters, such as moisture levels and skin temperature, smart prosthetic devices can proactively address the challenges posed by excessive sweating at the skin-prosthetic interface. Such devices facilitate timely adjustments to prosthetic fit and functionality, minimizing the risk of skin complications such as irritation and infection, thereby enhancing overall user comfort and device performance. Furthermore, incorporating AI and machine learning algorithms allows for the predictive analysis of hyperhidrosis episodes, enabling personalized, real-time interventions tailored to individual needs. This holistic approach acknowledges the psychosocial dimensions of hyperhidrosis, as excessive sweating can adversely affect self-esteem and social interactions, highlighting the importance of community support and mental well-being in the overall management strategy. However, to fully harness the potential of these technologies, it is crucial to address considerations regarding accessibility, affordability, and the ethical implications of data privacy and security in health monitoring. Collaborative efforts among researchers, manufacturers, healthcare providers, and policymakers are essential to ensure these innovative solutions are equitably distributed and effectively implemented. Ultimately, ongoing research and development in this field will not only lead to improved outcomes for individuals with hyperhidrosis but also contribute to a broader understanding of the role of technology in enhancing prosthetic care and promoting health equity across diverse populations.
